# Phage Encapsulation and Delivery Technology: A Strategy for Treating Drug-Resistant Pathogenic Microorganisms

**DOI:** 10.3390/ph18111688

**Published:** 2025-11-07

**Authors:** Yang Yue, Zhenbo Xu, Thanapop Soteyome, Mahesh Premarathna, Xiaomao Yin, Junyan Liu

**Affiliations:** 1 School of Food Science and Engineering, South China University of Technology, Guangzhou 510640, Chinamahesh.pr@nifs.ac.lk (M.P.); 2 Department of Laboratory Medicine, the Second Affiliated Hospital of Shantou University Medical College, Shantou 515051, China; 3 Home Economics Technology, Rajamangala University of Technology Phra Nakhon, Bangkok 10300, Thailand; thanapop.s@rmutp.ac.th; 4 Microbial Biotechnology Unit, National Institute of Fundamental Studies, Kandy 20000, Sri Lanka; yaner0722@hotmail.com; 5 Department of Laboratory Medicine, Guangzhou Red Cross Hospital, Jinan University, Guangzhou 510632, China; yinxiaomaoandy@sina.com; 6 College of Light Industry and Food Science, Guangdong Provincial Key Laboratory of Lingnan Specialty Food Science and Technology, Academy of Contemporary Agricultural Engineering Innovations, Zhongkai University of Agriculture and Engineering, Guangzhou 510225, China; 7 Key Laboratory of Green Processing and Intelligent Manufacturing of Lingnan Specialty Food, Ministry of Agriculture, Guangzhou 510225, China

**Keywords:** phage, delivery, encapsulation, CRISPR-CAS, medicine

## Abstract

Antimicrobial resistance (AMR) is one of the most critical challenges to global public health in the 21st century, posing a significant threat to healthcare systems and human health due to treatment failure and high mortality. The World Health Organization (WHO) estimates that, without effective interventions, AMR-associated infections could cause 10 million deaths annually and economic losses of up to 100 trillion US dollars by 2050. The rapid spread of drug-resistant strains, especially in hospital and community settings, has significantly reduced the efficacy of traditional antibiotics. With the continuous advancements in relevant research, bacteriophage (Phage) therapy is constantly innovating in the antimicrobial field. The application of frontier technologies, such as phage cocktails and engineered phages, has significantly enhanced the broad spectrum and high efficiency of phage therapy, which is gradually becoming a new generation of tools to replace antibiotics and effectively combat pathogenic bacteria. However, phage therapy is facing several challenges, including phage inactivation by gastric acid, enzymes, ultraviolet light, and mechanical stress, as well as the potential risk of bacterial phage resistance. Advanced encapsulation technologies such as electrospun fibers, liposomes, chitosan nanoparticles, and electrospray provide solutions to these problems by protecting phage activity and enabling controlled release and targeted delivery. This review addresses phage therapeutic studies of *Salmonella*, *Pseudomonas aeruginosa*, *Staphylococcus aureus*, *Escherichia coli*, and *Listeria monocytogenes*, summarizes the recent advances in phage research, and details the current development and applications of encapsulated phage technologies across various delivery modes.

## 1. Introduction

### 1.1. Pathogenic Microorganisms

Pathogenic microorganisms widely exist on the surfaces of objects frequently touched by human beings or in inadequately treated foodstuffs, becoming a potential health threat. These microorganisms can induce a wide range of diseases, such as skin inflammation, digestive disorders, or respiratory infections, thus posing a significant impact on individual health and public health. Their characteristics of spreading in the environment through direct contact, diet, or airborne transmission threaten human societies. *Salmonella* spp. is a widespread foodborne pathogen, a group of Gram-negative bacteria belonging to the Enterobacteriaceae family, and is one of the most important foodborne pathogens globally [1].

*Salmonella* is widespread in the natural environment, including soil, water, and the intestinal tracts of animals, and has become a common cause of food contamination and human infections because of its environmental adaptability and ability to spread [2]. The use of antibiotics in livestock may select for antibiotic-resistant bacteria, posing particular concern for the safety of our food supply chain. Antibiotic resistance, especially multidrug-resistant (MDR) Salmonella, is a major issue causing approximately 100,000 illnesses annually and is commonly associated with food from all possible contamination sources [3,4]. Antibiotic resistance in *Salmonella* has emerged as a major challenge in global food safety, with rising resistance rates closely linked to the overuse of antimicrobial drugs in livestock farming. As a key virulence mechanism, *Salmonella*’s ability to form biofilms enables the bacteria to evade host immune clearance and impede antibiotic penetration, significantly enhancing its environmental adaptability and pathogenicity. Furthermore, the presence of virulence genes (such as *invA*, *spvC*, *spvB1*, and *agfA*) further elevates the pathogenic potential of these strains. In a study of 38 *Salmonella* isolates, high resistance rates were observed against common antimicrobials, specifically: cefepime (60.5%), chloramphenicol (57.9%), trimethoprim-sulfamethoxazole (50.0%), amoxicillin-clavulanic acid (39.5%), azithromycin (28.9%), ofloxacin (26.3%), and cefuroxime (13.2%). Notably, 47.4% of these strains were identified as multidrug resistant (MDR), underscoring the severe risks posed by *Salmonella* resistance to public health and food safety [5,6,7,8]. However, it has now been shown that *Salmonella* is able to rely on the resistance nodulation cell division (RND) family of efflux pumps to efficiently efflux a wide range of antibiotics, such as chloramphenicol, using a proton motive force, which reduces the intracellular concentration of the antibiotics and diminishes their bactericidal efficacy. Together, these mechanisms allow *Salmonella* to survive antibiotic pressure and make treatment more difficult. Currently, regarding biofilms, *Salmonella* in mixed biofilms increases the risk of Salmonella contamination by enhancing biomass formation, resistance to disinfectants and food processing stresses, antibiotic tolerance, and human cytotoxicity [9]. This ability to form biofilms—involving bacterial aggregation and embedding within self-produced matrices—constitutes a key persistence mechanism for *Salmonella*, enabling its sustained survival in both environmental and host settings. For instance, non-typhoidal *Salmonella* serotypes can aggregate and embed within self-produced extracellular matrices to form so-called biofilms, posing significant challenges for microbial clearance [10,11].

*Staphylococcus aureus* is a Gram-positive bacterium which is frequently isolated from a variety of foods, such as egg products, vegetables, raw milk and dairy products, meat and seafood, and poses a significant threat to human health [12]. Methicillin-resistant *S. aureus* (MRSA) poses significant clinical and economic challenges due to increased incidence, prolonged hospital stays, and rising healthcare costs. For instance, a nationwide study in Japan estimated that MRSA infections result in over $2 billion in annual healthcare overspending and more than 14,000 deaths [13,14]. MRSA’s resistance is encoded by multiple genes it carries, which collectively confer a spectrum of resistance to β-lactam antibiotics, lincosamides, macrolides, clindamycin, and aminoglycosides [15]. The virulence of *S. aureus* is controlled by a complex regulatory system, including SigB (alternative σ-factor), Sar-family DNA-binding proteins, and the Agr group sensing system, which triggers severe infections mainly through a variety of toxins (e.g., haemolysins, PVLs, PSMs, EDINs, ETs, SEs, and TSST-1) [16,17]. *S. aureus* has been reported to be resistant to antibiotics mediated by *mecA* genes, VanA manipulators, *erm* genes, and modifying enzymes. Furthermore, colonization on implanted biomaterial surfaces begins with microbial surface component recognition and adhesion matrix molecules (MSCRAMMs). Subsequently, the biofilm matures through the synthesis of an extracellular polymeric matrix composed of host factors, polysaccharide intercellular adhesin (PIA), proteins, extracellular DNA, and lipids. This matrix effectively shields against host immune responses and antimicrobial agents, making infections difficult to eradicate. It also further enhances antibiotic resistance, rendering treatment increasingly challenging [18,19].

*Escherichia coli O157:H7* is an important foodborne pathogen capable of causing severe gastrointestinal diseases [20], and such cases are reported from contaminated food, undercooked beef, vegetables, dairy products, and tap water, all of which can contribute to the spread of the organism [21]. For *E. coli*, the extracellular matrix primarily consists of amyloid fibrils formed by amyloid proteins and polysaccharide cellulose fibers. The biofilm microenvironment of *E. coli* significantly promotes the evolution of gentamicin resistance, though adaptation pathways vary among different strains. Food-isolated strains achieve high-efficiency resistance through point mutations in key genes such as *aceE* and *atpG*, whereas reference strains rely on large-scale deletions in genes like *sbmA*, significantly altering cellular adaptability. These unique biofilm-specific characteristics serve as powerful drivers of bacterial evolution, promoting the emergence of antibiotic-resistant clones even in the absence of external pressure [22,23].

*Pseudomonas aeruginosa* is a Gram-negative bacterium widely distributed in the natural environment. Carbapenems are critical drugs for treating severe infections caused by multidrug-resistant pathogens. However, infections caused by carbapenem-resistant *P. aeruginosa* (CRPA) often respond poorly to most antimicrobial agents, with some strains exhibiting resistance to all available antibiotics. Of particular concern, certain CRPA strains can produce carbapenemases—enzymes that efficiently hydrolyze carbapenems and other β-lactam antibiotics—leading to treatment failure [24,25,26]. *P. aeruginosa* can be planktonic or sessile, and biofilms are typical of the sessile state, consisting of an extracellular matrix [27]. The pathogenicity of *P. aeruginosa* is primarily mediated by multiple virulence factors acting in concert, including exotoxins (such as ExoS and ToxA), elastase (LasB), and pyroxins (PhzM). These factors synergistically enhance the bacterium’s ability to invade host tissues and promote immune evasion. Furthermore, the bacterium can form biofilms on various surfaces, not only enhancing its environmental survival but also significantly increasing its resistance to conventional disinfection methods [28]. *P. aeruginosa* also expresses a variety of efflux pumps, such as MexAB-OprM, MexXY-OprM, etc., which excrete β-lactam, fluoroquinolone, and aminoglycoside antibiotics [29]. In addition, β-lactam resistance in *P. aeruginosa* can arise through a combination of mechanisms such as altered PBP3 target proteins, reduced pore protein uptake, increased antibiotic efflux, β-lactamase degradation, horizontal gene transfer, and metabolic changes [30]. *P. aeruginosa* forms biofilms by adhering to surfaces and secreting extracellular matrix, undergoing multiple stages including adhesion, proliferation, maturation, and dispersion. This structured microbial community effectively resists antibiotics, oxidative stress, and host immune attacks, leading to persistent chronic infections and inflammation in medical devices and the lungs of cystic fibrosis patients. The final dispersal step of biofilms can also lead to systemic dissemination of infection, posing a major challenge for clinical treatment [31,32].

*Listeria monocytogenes* is a deadly foodborne pathogen commonly found in food processing facilities and can cause a zoonotic disease. Milk and dairy products are considered excellent substrates for the development of *L. monocytogenes* [33]. *L. monocytogenes* is a severe foodborne illness with a mortality rate ranging from 20% to 30% [34]. Its cold tolerance and ubiquitous nature make it prone to contaminating food, particularly ready-to-eat (RTE) products [35]. Numerous *L. monocytogenes* outbreaks have also been linked to RTE salads, fruits, and vegetables, whose consumption is increasingly high and globally widespread. Antibiotic resistance in bacteria has become a growing concern due to excessive antibiotic use in clinical settings over the past decades [36]. *L. monocytogenes* has been reported to exhibit resistance rates of 66.7% to clindamycin, 70% to penicillin G, 50% to amoxicillin, and 55% to vancomycin [37]. In the United States, its outbreaks cause an economic burden of more than $2 billion per year [38]. *L. monocytogenes* significantly enhances its survival and persistence in food processing environments by forming biofilms. Biofilms provide physical protection, enabling bacteria to withstand routine cleaning and disinfection protocols. This structure also confers substantial adaptive antibiotic resistance, with biofilm-associated infections often requiring 10 to 1000 times higher antibiotic doses than planktonic bacteria. This poses severe challenges for food safety and clinical treatment [39,40].

### 1.2. Antimicrobial Resistance

With increase in antimicrobial resistance, antimicrobial resistance (AMR) is now a chronic public health problem worldwide, with 10 million deaths projected annually by 2050. The increase in AMR is mainly due to the overuse and inappropriate application of antimicrobials, especially antibiotics, which accelerates the evolution and spread of drug-resistant strains of bacteria [41]. A study investigated the cases of infections in Ethiopia from 2015 to 2024 and found that *Acinetobacter baumannii* infections accounted for 9.13% of bacterial infections, with multi-drug resistant (MDR) strains as high as 88.22%, and resistance to antibiotics, such as cefotaxime, was 83.18%, with a higher prevalence especially in cases of sepsis [42]. Another study showed that carbapenem-resistant *Klebsiella pneumoniae* (CRKP) accounted for 14.2% of bloodstream infections (BSIs) and A. baumannii resistance was as high as 87.8% of BSIs in Brazil during the period 2022–2023, and that mortality rates of patients infected with resistant strains were significantly higher than those of non-resistant strains [43]. In the classification of AMR, MDR, and extensive drug resistance (XDR) represent the severity of resistance; MDR refers to bacteria that are resistant to at least three classes of antibiotics, whereas XDR refers to bacteria that are highly resistant to almost all classes of antibiotics, retaining susceptibility to a very small number of drugs, with a range of resistance that extends far beyond MDR [44]. In the case of *P. aeruginosa*, for example, the prevalence of its MDR strain exceeds 40% in Latin America and reaches 28.4% in Europe, significantly increasing the complexity of infection treatment [45]. An analysis of mortality rates and disability-adjusted life years (DALYs) for all age groups and specific age groups from 1990 to 2021 shows that drug-resistant bacteria impose a significant burden on people of all ages, so action must be taken to find alternative treatments to antibiotics [46,47].

### 1.3. Materials and Methods

Initially, we retrieved a substantial number of publications—approximately 50 of the most relevant documents per keyword category, amounting to a total of around 200 documents. We then reviewed the abstracts of these articles and conducted a preliminary screening to identify literature aligned with the thematic focus of our review, resulting in roughly 100 selected papers. Subsequently, we examined the methodology and conclusions of the research articles and performed a comprehensive overview of the review papers. By integrating these findings with the current state of research, we identified emerging research hotspots and gaps, which we synthesized into a coherent logical structure to form the preliminary framework of the review.

We anchored the framework around three core keywords: ‘phage cocktail’, ‘CRISPR-Cas’, and ‘phage encapsulation and delivery’. For each keyword, we conducted a more detailed literature search covering the past six years (2019–2025), retrieving over 100 articles per category and accumulating approximately 400 publications in total. After a preliminary assessment of the abstracts and conclusions, we closely examined around 100 papers focusing on current research hotspots. More than half of these were selected to develop the framework, supplemented with critical reflections, leading to the initial draft of the manuscript.

**Figure 1:**
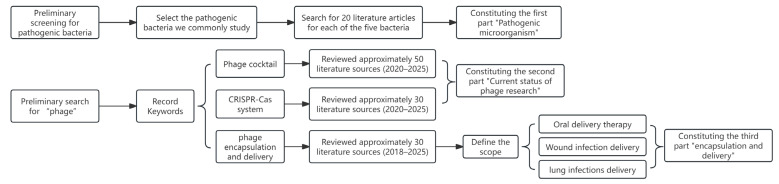
Flowchart of the article screening process, including keywords, number of articles read, and number of final citations.

Nevertheless, we recognized that the coverage remained somewhat limited. To enhance the depth of the review, we further refined the search strategy using additional keywords such as “phage oral delivery”, “phage skin delivery”, “phage lung delivery”, and “cocktail of more than two phages”. Concurrently, we restructured the overall organization of the article and incorporated critical appraisal to strengthen its reflective and logical qualities. The final version of the review cites 160 references. Details are shown in Figure 1.

## 2. Current Status of Phage Research

**Figure 2:**
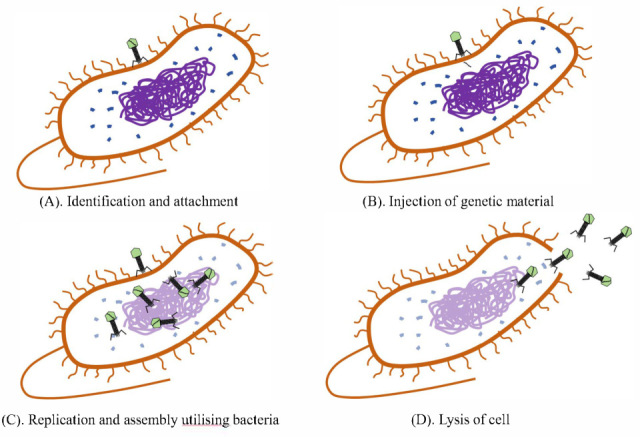
The principle of bacteriophage action. The figure depicts the four stages of phage infection of bacteria. When phages reach sufficient numbers, they trigger lysis, leading to bacterial death. Throughout the entire figure, the purple lines represent the genetic material DNA, the blue lines depict ribosomes, and the orange lines indicate the bacterial cell wall, fimbriae, and flagella.

Bacteriophages (Phages) are viruses that can specifically infect and replicate within a target bacterium, eliminating 4–50% of bacteria [48]. Phage therapy has re-entered the scientific horizon as a precise and efficient biological approach. Phage therapy is flexible in that it can be personalized to different patients, greatly increasing the efficiency of treatment [49]. Currently, phage therapy programs have been established in the United States, Belgium, France, and Sweden, and there are long-term programs in the Republic of Georgia and Poland [50]. The phage lytic cycle is the process by which phages replicate within bacterial cells and eventually cause cell lysis. The cycle begins with phage attachment to the host cell surface and is divided into two phases, reversible and irreversible. Initially, the phage establishes transient contact with the bacterial cell surface through reversible attachment. Subsequently, the phage enters the irreversible attachment phase by securely binding to a specific receptor on the host cell surface. Following this binding, the phage releases lytic enzymes that degrade the host cell wall, thereby injecting the phage’s genetic material into the host cell. The phage genome takes over the host cell’s metabolic system and redirects it towards phage DNA replication and protein synthesis to produce new phage particles. At the same time, the host cell’s genome is degraded, and phage components are assembled within the cell. Eventually, the host cell undergoes lysis, releasing new phage particles that can infect other cells as shown in Figure 2. The key to the lytic cycle is the action of phage proteins and endolysins, which disrupt the host cell membrane and degrade the cell wall, respectively, leading to cell lysis in response to osmotic imbalance [51,52].

Phages, with their unique bacterial targeting and self-replicating ability, are able to efficiently lyse drug-resistant strains without disturbing the host microbiota. It has been found that phage SEP4 has a significant inhibitory effect on multidrug-resistant *Salmonella* and its biofilm formation on food matrices, especially at 25 °C. In addition, phage SEP4 was able to completely inhibit the mature biofilm on the surface of lettuce leaves and chicken meat within 4–6 h [53]. However, prolonged use of phages can likewise cause strains of bacteria to become resistant to phages, so it is important to keep developing phages in conjunction with some of the currently popular technologies to meet the human need for phage therapy.

### 2.1. Phage Cocktails

Phage cocktails, which are mixtures of multiple phages, can target a wide range of bacterial strains (Table 1). There are three types of phage cocktails: multi-species phage cocktail, single-species phage cocktail, and single-strain targeted phage cocktail [54].

#### 2.1.1. Application of Phage Cocktails

Pelyuntha designed a single-strain phage cocktail, a phage cocktail specifically targeting the species *Salmonella*. Due to the diversity of *Salmonella*, it is difficult for a single phage to fully eradicate this microorganism. By combining three *Salmonella* phages, the cocktail broadens the lysis range, enhances coverage of Salmonella’s diversity, and overcomes the narrow host range limitation of a single phage [55]. As an alternative to antibiotic therapy, a mixture of three phages was significantly more effective than one phage alone in lysing *E. coli* against *E. coli* resistant to the antibiotic-resistance gene *mcr-1* [56]. Guo developed a novel phage, PHC-1, comprising three phages, JDYN, JDF86, and SLPW, in a 1:1:1 ratio, which expands the host range compared to a single phage in the fight against drug-resistant *S. aureus* in bovine mastitis, and provides a safe and efficient alternative to antibiotics in the treatment of bovine mastitis [57]. The intestinal flora is highly complex. Liu found that administering a phage cocktail via oral gavage to piglets’ intestines actively forms a protective barrier, altering the microbial community composition in a way that promotes intestinal health [58]. Shiue et al. developed a phage cocktail hydrogel dressing incorporating three distinct phages, each specifically lysing its target bacterial host without affecting non-target species, thereby constituting a multi-species phage cocktail [59]. Similarly, Xue et al. selected three pathogenic bacteriophages and formulated them into a phage cocktail for the treatment of bovine mastitis. This multi-species cocktail broadens the treatment spectrum, enhancing the prevention and control of polymicrobial infections on dairy farms [60].

#### 2.1.2. Progress in Design Methodology Research

The design of the phage cocktail is also being innovated. Królikowska developed a systematic phage cocktail production method using anti-mastitis treatment as an example, integrating bioinformatics, including phage genome and receptor protein analyses, lytic activity testing, and resistance assessment, which markedly improved the effectiveness and relevance of the phage cocktail [61]. Hegarty proposes an advanced phage cocktail design approach through integrated modeling, genomics, and experimental validation. The core lies in predicting phage-host interactions, optimizing phage combinations, and assessing environmental impact. The method is more systematic than traditional trial-and-error methods and is a more efficient solution for designing phage cocktails [62]. Kunisch screened two efficient phages out of four and trained them in a targeted manner to develop a phage cocktail production method with enhanced antibacterial and anti-biofilm effects [63]. Li investigated a new method for the rapid construction of an efficient phage cocktail using phage-resistant mutant strains, combining the effective new phage Va3 with the initially used Va1 phage to synthesize a phage cocktail that greatly improved phage lysis [64]. Artificial Intelligence (AI) has grown rapidly in recent years, and its great advantage is the availability of rich, high-quality data for building machine learning models [65]. For example, predicting phage-host relationships and predicting host range design phage cocktails [66,67,68]. Smug predicted a scenario in which four phages infested *Klebsiella pneumoniae*, with KP34 and KP15/KP27 phages mixed in a 1:1 ratio, were more effective than a single phage [69]. Designing more efficient phage cocktails by combining different phage combinations with different effects on targeted elimination of microorganisms has become a powerful tool for phage cocktails [70].

### 2.2. Engineered Phages Based on the CRISPR-Cas System

Engineered phages are chemically modified and genetically altered to enhance phage host range, effectiveness, and safety and to impart new functions, including detection and antimicrobial therapy. There are four main types of phage engineering: chemical modification, in vivo genetic engineering, in vitro synthesis of phage genome, and CRISPR-Cas-based phage engineering [71]. Currently, the most prominent research is engineering phages using the CRISPR-Cas system. The CRISPR-Cas system is an adaptive immune system for prokaryotes that identifies and eliminates invading phages or plasmids by storing exogenous DNA fragments [72]. The mechanism of action of the CRISPR-Cas system is divided into three processes: adaptation, expression, and interference. The first process involves recognizing the exogenous DNA fragments and integrating them into CRISPR sequences to form new spacers. These sequences undergo transcription to generate pre-crRNA, which is further processed into mature crRNA. The crRNA then guides the Cas nuclease to recognize and cleave exogenous nucleic acids complementary to the spacer sequence, thus resisting their invasion [73,74]. Although the CRISPR-Cas system is said to be a defense system for prokaryotes, it can also be used to attack prokaryotes. There are different types of CRISPR-Cas systems, which are divided into two broad categories. Class 1 CRISPR-Cas systems (types I, III, and IV) encode multisubunit effector complexes. In contrast, class 2 CRISPR-Cas systems (types II, V, and VI) use only a single-subunit effector protein [75]. Engineered phage based on the CRISPR-Cas system operates primarily from two approaches: one is to modify the tail gene of phage through CRISPR-Cas9 to broaden the host range of phage; the other is to deliver the CRISPR-Cas system that can knock out the drug-resistant genes of the host through phage or directly deliver the phage to kill certain genes of the host. The CRISPR-Cas system is used to kill certain genes of bacteria, so that the bacteria die directly. The most common is the use of type I and type II CRISPR-Cas systems [76,77].

#### 2.2.1. Engineered Phages Based on Type I CRISPR-Cas System

Among CRISPR-Cas systems, type I systems are characterized by the presence of the distinctive nuclease Cas3 and are the most abundant and diverse CRISPR-Cas systems in nature. Type I systems display a complex structure, comprising conserved adaptation modules (Cas1 and Cas2), as well as multiprotein effector complexes containing effector modules of the characteristic nuclease Cas3 and constituting cascades [78]. Gencay utilized *E. coli*’s type I-E CRISPR-Cas system to design a new CRISPR-Cas system combined with an engineered phage. The phage’s tail was altered through gene editing, and CRISPR sequences with Cas nuclease targeting *E. coli* were incorporated into the phage’s genetic material, forming CRISPR-Cas-armed phages (CAPs). Four optimal CAPs were obtained through validation screening, and they were integrated into a phage cocktail named SNIPR001, which combines the most advanced phage screening with phage tail fiber engineering and the CRISPR-Cas system and opens up a new avenue for phage therapeutics [79].

#### 2.2.2. Engineered Phages Based on Type II CRISPR-Cas System

Type II CRISPR-Cas is considered to be the most compact CRISPR-Cas system and the most widely used CRISPR-Cas gene editing system, which can perform targeting and cutting functions with only a single effector protein Cas9, and has applications in gene editing, knocking out drug-resistant genes, and genetic modification, and it is a pair of scissors for genetic engineering [80,81,82,83]. Using a type II CRISPR-Cas9 system, Park developed an antibacterial approach targeting the *nuc* gene of *S. aureus*, which was able to recognize the PAM sequence next to the *nuc* gene, cleave the genomic DNA, and trigger bacterial death. Additionally, 10 superantigens and 11 cytotoxic genes of the host were eliminated to improve safety after lysing the bacteria, and expanding the host by tail fibronectin modification scope [84].

However, the therapeutic efficacy of phages is often compromised by environmental vulnerability during administration. This critical limitation has driven the rapid development of advanced encapsulation and delivery technologies, which we will explore in the following sections.

## 3. Recent Research Advances in Phage-Based Encapsulation and Delivery

**Figure 3:**
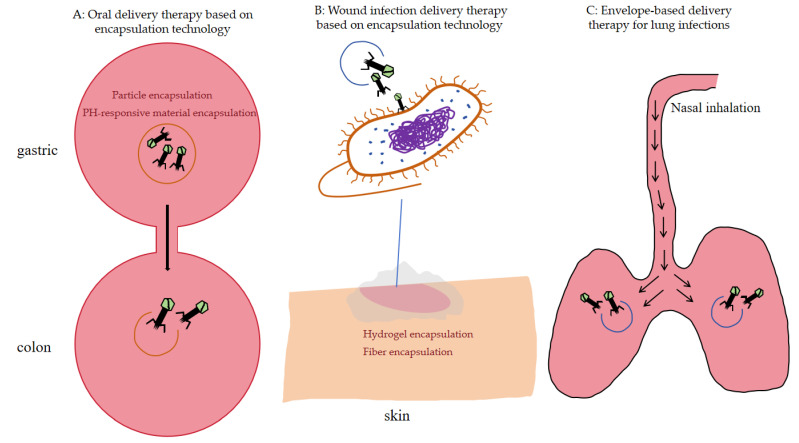
Phage encapsulation and delivery. The figure illustrates three phage delivery pathways: As shown in section (**A**), encapsulated phages successfully traverse the gastric environment to reach the intestinal tract for release. As shown in section (**B**), encapsulated phages enable sustained release at wound sites. As shown in section (**C**), encapsulated phages enter the lungs via inhalation, where release achieves pathogen eradication. In (**A**,**C**), the circles enveloping the bacteriophages represent the encapsulation material. In (**A**), the entire pink outer circle represents different sections of the gastrointestinal tract. (**B**) depicts the skin wound and the bacteriophage dressing applied to its surface. (**C**) represents the lungs, with the arrows indicating the delivery pathway of the bacteriophages during the inhalation process.

Encapsulation entails the use of polymers, proteins, or other materials to create a continuous layer of membranes, gels, microcapsules, films, or fibrous webs that encapsulate the active substance internally or embed it in a matrix by physical or chemical means [85]. Phages exhibit very low environmental tolerance and can be rendered non-functional by denaturation, internal DNA damage, and internal structural changes caused by harsh environments. Therefore, it is necessary to use some strategies to protect the phage from performing its functions when necessary. Membrane embedding technology is very effective in protecting phages, such as sodium alginate membrane, cellulose acetate membrane, chitosan membrane, PCL membrane, electro discharge fiber embedding and microcapsule protection, which have been used in food packaging to reduce the number of E. coli, *Salmonella*, *P. aeruginosa*, *L. monocytogenes*, and *Vibrio parahaemolyticus* in foods to a large extent, reducing the chances of foodborne pathogens attacking the body at the source of infection (Table 2) [86]. In medical phage therapy, encapsulation protects the phage, preserves its biological activity, and enhances therapeutic efficacy [87]. Currently, encapsulation technology is constantly being optimized, and mainstream research is divided into three main areas: improving the encapsulation rate, enhancing the performance of encapsulated phage, and developing new encapsulation materials [88]. Currently, there are three main routes of drug delivery, as shown in Figure 3.

### 3.1. Optimization of Traditional Encapsulation Techniques

Traditional encapsulation techniques have significantly improved the efficiency and stability of phage encapsulation by optimizing the material ratio and preparation process. In the context of the ongoing research and development of encapsulation materials, Koçer carbonated the amount of sodium alginate for encapsulation of *Salmonella* phage on the basis of sodium alginate membranes, and found that the highest encapsulation rate of *Salmonella* phage was achieved when the ratio of encapsulating material to phage was 2:1 [89]. Another classical strategy, liposome encapsulation, is favored because it mimics the structure of the cell membrane [90]. Choi’s research also encompassed the investigation of a co-encapsulation technique, wherein the optimal ratio of a mixed system comprising maltodextrin and alginate was determined for the material of this technique. It was observed that this ratio not only improved the encapsulation rate after encapsulation of the T4 phage, but also resulted in the phage having a high lysogenic activity and strong antimicrobial activity [91]. Guo optimized the conditions for the preparation of phage SaTp-04 CA-SA membranes using casein (CA) and sodium alginate (SA) as substrates and improved the encapsulation rate of SaTp-04 [92]. The encapsulation rate of phage has been enhanced through the utilization of conventional encapsulation technology. This development has been instrumental in achieving stable encapsulation of phage, thereby markedly enhancing its efficiency in various delivery modalities, including oral, topical, and inhalation methods. This advancement serves as a crucial foundation for the effective implementation of medical phage therapy.

### 3.2. Oral Delivery

Gastrointestinal infections represent the most prevalent infectious diseases, with an estimated 1.9 million deaths worldwide each year due to gastrointestinal diseases. Oral administration represents a highly convenient method for drug delivery, allowing patients to administer the medication themselves. However, a significant challenge in orally delivering therapeutics targeting the intestinal tract is the necessity to traverse the harsh gastric environment, characterized by hydrochloric acid and digestive enzymes, such as pepsin, which facilitates food digestion and eliminates pathogenic bacteria. The gastric pH typically ranges from 1.5 to 3.5 [93]. To ensure effective oral delivery, one of the problems that needs to be solved is the need for acid-resistant encapsulation materials to encapsulate the phage, so that it can successfully pass through the strong acidic environment of gastric acid to reach the intestinal tract and perform its function.

#### 3.2.1. Particle Encapsulation

Particle encapsulation refers to the physical or chemical encapsulation of a phage in the interior of tiny particles to protect the phage [94,95]. The encapsulated phage exhibits enhanced acid resistance, enabling effective delivery to the gastrointestinal tract for therapeutic purposes. Elsayed developed chitosan nanoparticles (CS-NPs) for encapsulation of MRSA phage, a material that protects the phage from acidic environments and enzymatic damage [96]. They prepared chitosan nanoparticles (CS-NPs) of phage HK6 by cross-linking chitosan with sodium tripolyphosphate (TPP) using an ionic gel method, which can achieve a high encapsulation efficiency of 97%. Temsaah further demonstrated that CS-NPs encapsulated phage HK6 exhibited stronger antibacterial activity compared to conventional chitosan-encapsulated phage, especially in the MRSA infection model [97]. Phages encapsulated in CS-NPs benefit from a more resilient protective coating than those encapsulated in agarose, alginate, or latex. Karthika introduced a phage mixture encapsulated in a chitosan-based matrix to address the limitation of single phage encapsulation, which also allows effective delivery to the gut for the treatment of gastrointestinal disorders beyond the strong acidic environment [98]. The advantages of this approach are the introduction of a phage cocktail combinatorial strategy to broaden the antimicrobial spectrum and reduce the risk of resistance, the bead-like structure suitable for gastrointestinal delivery, and the high efficiency verified by in vitro biofilm removal experiments. This particle packaging creates a protective barrier for the phage, not only in the gastric acid environment, but also for other applications such as topical administration and cryopreservation, offering versatile solutions for phage delivery. However, existing studies are subject to several key limitations. Although the encapsulation efficiency (EE%) is widely reported, neither the phage release kinetics nor the underlying release mechanisms have been quantitatively characterized. For instance, Karthika’s study asserted effective penetration in a strongly acidic environment; however, it did not provide cumulative release profiles at intestinal pH levels, which are essential to determine whether burst release or premature leakage occurred. Current encapsulation strategies primarily rely on the passive dissolution and diffusion of chitosan-based systems, lacking deliberate design and investigation into active, stimuli-responsive controlled release. Future research should therefore prioritize a mechanistic understanding of particle encapsulation, with emphasis on release rates and trigger mechanisms.

#### 3.2.2. PH-Responsive Material Encapsulation

The disadvantages of particle encapsulation can be effectively avoided with pH-responsive materials, which are chosen to dissolve or swell under colonic pH conditions as the ideal material for achieving colonic-targeted delivery [99,100,101]. In contrast to basic phage protection, pH-responsive materials enable targeted release of phages in specific pH environments, optimizing their therapeutic efficacy. Pectin is not digested in the stomach, but can be degraded by pectinase secreted by intestinal bacteria to produce short-chain fatty acids, while releasing encapsulated material in the colon, making it a good pH-responsive encapsulation material [102]. A groundbreaking “infect first, encapsulate later” strategy was proposed. Rather than directly encapsulating the fragile bacteriophages, they employed pH-responsive polymers (methacrylic acid-ethyl acrylate copolymer) to encapsulate bacteria that had already been successfully infected by the phages [103]. Alginate also serves as a robust pH-responsive material; the pKa values of β-D-mannuronic acid and α-L-guluronic acid on the glycosyl chain of sodium alginate are higher than the pH of the gastric juice, and when it reaches the colon, the pKa values are lower than the pH, which results in the dissolution of the sodium alginate gel. Encapsulation of phage by dropwise addition of sodium alginate solution to calcium chloride solution to form microspheres to provide acid-resistant protection is a simple process, but a single material may limit protection in more extreme environments [104]. Dlamini found that the addition of calcium carbonate to alginate-carrageenan encapsulation prolonged the active duration of phage encapsulated in alginate-carrageenan microcapsules in acidic conditions. This technology forms a physical barrier that protects phage from pepsin degradation, allowing for a large amount of phage to be released in the intestinal environment, making it suitable for oral treatment of intestinal infections such as *Salmonella* enteritis [105].

The considerable pH variation between the colon and the stomach, while somewhat improving release rates compared to particle encapsulation systems, introduces a series of challenges. For pectin-based encapsulation materials, their relatively low mechanical strength makes them prone to rupture in the acidic gastric environment, leading to premature phage release [106]. Similarly, certain copolymers can cause abrupt phage release rather than sustained delivery, thereby failing to achieve continuous therapeutic effects. Moreover, the degradation of these polymers may cause intestinal damage—an aspect overlooked in the cited study [107]. Similarly, certain copolymers can cause abrupt phage release rather than sustained delivery, thereby failing to achieve continuous therapeutic effects. Moreover, the degradation of these polymers may cause intestinal damage—an aspect overlooked in the cited study [108]. An even more critical issue with pH-responsive materials lies in the similar pH ranges of the small intestine (pH 6–8) and the colon (pH 5–7.5). Since many responsive materials initiate release at pH 6.8, a substantial proportion of phages may be released prematurely in the small intestine, resulting in insufficient dosage reaching the colon [109]. Therefore, more refined material designs are necessary to overcome this limitation and enable precise site-specific release.

Different encapsulation methods emphasize different aspects depending on the intended purpose. For broad-spectrum inhibition of pathogens throughout the gastrointestinal tract, robust particulate encapsulation is more suitable. For targeted treatment of colonic diseases, further optimization of pH-responsive materials is required, or the development of materials with multiple response mechanisms such as enzyme-responsive or time-responsive properties.

In contrast to the systemic challenge of gastrointestinal delivery, topical management of localized wound infections demands a different set of characteristics from the delivery system, primarily focusing on biocompatibility and sustained release at the site of infection.

### 3.3. Wound Infection Delivery

Wound infections are frequently caused by drug-resistant organisms such as *P. aeruginosa* or MRSA. Phage encapsulation improves therapeutic efficacy through topical delivery, and wound therapy is the most common application of phage therapy. In contrast to oral delivery, wound delivery generally places a higher value on the adherence and biocompatibility of encapsulated materials.

#### 3.3.1. Hydrogel Encapsulation

As wound dressings, hydrogels are a favorite material for scientists to consider, and various hydrogel materials have been employed for phage encapsulation. Mukhopadhyay developed a binary temperature-sensitive hydrogel consisting of Planctin F-127 (PF-127) and hydroxypropyl methylcellulose (HPMC), which stabilizes phages and mucins. PF-127 and HPMC binary temperature-sensitive hydrogel achieves stable encapsulation and high encapsulation efficiency of phage and mucin through the temperature-sensitivity of PF-127 and hydrophilic network of HPMC, which is suitable for wound site administration. This encapsulation technology. This formulation resulted in a phage-activated scaffold (PAS) wound dressing that effectively treatment of wound infections caused by MDR-*Acinetobacter baumannii*, which significantly improves the antimicrobial efficiency, inhibits biofilms, and promotes wound healing [110]. Abed engineered a novel hydrogel based on SA, CMC, and HA, integrating for the first time the antimicrobial effect of SAM-E.f 12 phage and the pro-healing properties of HA to achieve efficient antimicrobial and rapid healing of Enterococcus faecalis infected wounds. It resolves the challenge of mutual constraints between antimicrobial effects and tissue regeneration in traditional therapies, representing the integrated future direction of wound treatment [111]. Khazani formulated a composite hydrogel consisting of SA, CMC, and HA for stable encapsulation and sustained release of phage through optimized ratios and Ca^2+^ cross-linking for enhanced wound infection therapy. The use of Ca^2+^ to form an ionic cross-linking network with SA increases the mechanical strength and elasticity of the hydrogel, allowing it to withstand the mechanical stresses of the wound site and the wet environment [112].

As wound dressings, the most critical performance parameter is adhesive strength. Although all three evaluated dressings exhibit sufficient mechanical strength to withstand skin stretching and folding without damage, their adhesive properties have not been systematically investigated. For instance, clinically available hyaluronic acid (HA) hydrogels are known for their poor bioadhesion, which often leads to inadequate fixation and thus significantly compromises therapeutic outcomes [113]. Additionally, hydrogels are generally costly to produce and typically require large-scale manufacturing. To address these challenges, researchers could explore alternative strategies such as single-step cross-linking or 3D printing technologies to streamline the fabrication process [114,115].

#### 3.3.2. Fiber Encapsulation

Electrospun fiber encapsulation is a method of encapsulating active substances in nanofibers by electrospinning technology. It is divided into uniaxial electrospinning and coaxial electrospinning. Uniaxial electrospinning involves blending a polymer solution with phages and extruding them into fine fibers under a high-voltage electric field, ensuring uniform phage distribution throughout the fiber matrix. In contrast, coaxial electrospinning utilizes coaxial needles to spray two kinds of solutions at the same time, one is the mix of polymer solution and phage, and the other is the shell layer solution, which forms the fiber of the core–shell layer structure [116,117]. Liu developed a novel electrospun fiber encapsulation material that embeds anti-*P. aeruginosa* phage (Neko) into nanofibers by uniaxial and coaxial electrostatic spinning, which can be applied directly to infected wounds with sustained release of the active phage, providing a dressing solution for the treatment of infected wounds [118]. Using coaxial electrospinning technology, phages were encapsulated within the core of the fiber while protected by a PVP shell layer, and nanofibres with a core–shell structure were successfully prepared. The rapid dissolution, biocompatibility, and mechanical properties of PVP make it an ideal material for wound dressing. Electrospun fibers offer high flexibility in local delivery, but the preparation process is complex and costly. Coaxial electrospinning provides better shell layer protection than uniaxial, but the rapid solubility of PVP may lead to excessive release.

Moving from external wounds to internal respiratory infections, the delivery paradigm shifts again, requiring technologies that can navigate the complex architecture of the lungs to achieve deep tissue deposition.

### 3.4. Pulmonary Delivery

Respiratory infections, especially infections caused by drug-resistant bacteria, have always been a challenge to treat due to limitations in the way treatments are administered. In 2019, an estimated 1.5 million deaths globally were attributed to respiratory infections caused by drug-resistant pathogens [119]. The key challenge in treating lung biofilm infections is drug delivery; intravesical phage administration is commonly used but predicts poor biodistribution [120]. Whereas phage encapsulation for respiratory therapy is currently of two main types: inhalation therapies utilizing dry powder formulations and those employing nebulized liquid phage formulations.

#### 3.4.1. Powder Inhalation Therapy

The liquid mixture of phages and encapsulated material is atomized into small droplets by spray drying and spray freeze drying, followed by solvent evaporation through hot air or lyophilization to produce dry powder particles. These particles are delivered deep into the lungs using some techniques, such as a dry powder inhaler, to achieve therapeutic effects [121]. Sawant developed a liposome encapsulation system for *P. aeruginosa* phage, using thin film hydration to encapsulate the phage in liposomes, showing the promising potential of inhaled phage liposomes in the treatment of lung infections caused by *P. aeruginosa* [122]. Liu investigated an inhaled PLGA porous microsphere dry powder formulation (PMPs-PI), which achieved efficient synergistic treatment of MRSA pneumonia by combining heat-resistant phage NKU-1 with ICG photothermal therapy [123]. This design achieves the integration of physical encapsulation protection with physicochemical synergistic therapy. The microspheres themselves shield the phage and ensure its efficient pulmonary deposition, while photothermal therapy and phage therapy generate a powerful synergistic effect, offering a novel non-invasive combined treatment approach for deep-tissue MRSA pneumonia. Liposomes exhibit high deposition efficiency in pulmonary delivery and can be stored for long periods of time, but they are expensive to prepare and suitable for mass production.

#### 3.4.2. Nebulized Inhalation Therapy

The design of liquid nebulization is much simpler compared to dry powder production, which requires further drying after liquid nebulization and is more demanding in terms of encapsulation materials. Nebulized liquid formulations bypass the drying phase, making them a more convenient inhalation therapy modality. Li innovated an inhaled liposome-phage K nanocomplex in which he nebulized a solution of liposome-phage K liquid mixture into an aerosol via a vibrating mesh nebulizer for pulmonary drug delivery to target Staphylococcus aureus infections. The EPC-phage K formulation exhibited superior nebulization stability, lung deposition, and intracellular antimicrobial efficacy [124]. Cao optimized the liposome formulation by applying liposome encapsulation technology to the nebulized inhalation delivery of phage. By studying both jet and vibrating mesh nebulizers, he highlighted the critical role of liposomes in protecting phage from the mechanical stresses of nebulization and found that vibrating mesh nebulizers were better at achieving efficient lung deposition [125]. These studies have elevated liposomes from simple protective carriers to highly efficient delivery platforms, overcoming the longstanding technical challenges of poor stability and inadequate pulmonary distribution in liquid phage aerosol formulations. This breakthrough paves the way for the clinical translation of inhalation therapies. The electrospinning technology mentioned in the previous section can also be nebulized and designed for the treatment of respiratory tract infections. Liquid atomized encapsulated products have poor storage stability and should not be kept for long periods of time, but because of their simple process, it is more suitable for small-scale rapid development.

For commercial products requiring long-term storage and distribution, powder inhalation is the inevitable direction, but its core lies in developing mild drying protection technology. For individualized treatments prepared on-site in hospitals, optimizing nebulized formulations and protective agents represents a more practical and flexible approach.

### 3.5. Novel Encapsulation Material Breakthrough

As encapsulation technology continues to evolve, innovative materials have been explored to enhance phage delivery. Liu identified a natural material that can withstand UV, developing sporopollenin exine capsules (SEC) derived from plant pollen grains. This novel encapsulation material, for the first time, effectively shielded phages from UV-induced damage [126]. Sporopollenin is one of the most durable materials known in nature. This study demonstrates for the first time that SEC provides exceptional UV protection for phages, thereby resolving the core challenge of rapid inactivation caused by sunlight exposure in outdoor applications such as agriculture and environmental disinfection. This breakthrough paves the way for a new “nature-inspired” approach to phage-based biopesticides [127]. Nano-emulsion, a colloidal dispersion system within the realm of nanotechnology, has also been utilized. Kaur developed a novel nano-emulsion encapsulated material to disperse phage in a system of ultra-small droplets, which facilitates wound washing and enhances phage-bacteria contact efficiency through atomization design [128]. Natural products have also been used in this field. Wdowiak employed green tea extract as a reducing and stabilizing agent to synthesize environmentally friendly nanoparticles (G-TeaNPs), suitable for complex wound environments [129]. Easwaran combines liposome technology with phage mixtures to form L-PCT, providing a novel treatment for postoperative wound infections caused by multidrug-resistant bacteria MDR [130].

### 3.6. Application of Simulation and Prediction Techniques to Phage Encapsulation

Currently, more researchers in this field are using simulation and prediction techniques to simulate and predict the stability and release behavior of phage in different environments (e.g., the gastric acid environment). Elsayed’s study developed chitosan nanoparticles (CS-NPs) to encapsulate phage and tested their stability in simulated gastric fluid (SGF, pH 3), and the results showed that CS-NPs encapsulated phage could effectively resist enzymatic destruction by pepsin and significantly improve the ability to tolerate gastric acid [96]. Dlamini’s study evaluated the protective effect of sodium alginate with κ-carrageenan (ALG-CG) and a composite containing calcium carbonate (ALG-CG-CA) by simulating the gastrointestinal environment, and found that the recovery rate of phage encapsulated by ALG-CG-CA was significantly higher than that in an acidic environment. ALG-CG or unencapsulated phage, and could extend the duration of phage activity and provide more uniform protection [105]. Zhang et al. conducted simulated gastric environment tests on composite microcapsules of xanthan gum (XG), sodium alginate (SA), calcium chloride (CaCl_2_), and chitooligosaccharides (COS) to optimize ratios to enhance the stability and release efficiency of phage in an acidic environment [131]. Jamaledin et al. developed a predictive model using computational fluid dynamics (CFD) technology to optimize the phage encapsulation of polylactic acid-hydroxyacetic acid copolymer (PLGA) microspheres, comparing two methods: direct encapsulation by double emulsification technology (W/O/W) and preparation of empty microspheres followed by encapsulation by adsorption or osmotic encapsulation (post-encapsulation), and found that post-encapsulation was more optimal for in vivo release, showing higher [132]. Tao’s team designed a bio-responsive hydrogel (Gel-ADH and DF-PEG matrix) and validated its encapsulated phage cocktail (FPR-30) against *P. aeruginosa* infection in an ex vivo human skin model to simulate the wound environment, demonstrating that the hydrogel can release phage on-demand when triggered by bacterial secretion enzymes or pH changes, significantly prolonging the duration and improving the stability for localized wounds. This demonstrates that the hydrogel can release phage on demand, triggered by bacterial secretory enzymes or pH changes, significantly prolonging the duration of action and improving stability, providing an innovative solution for the treatment of localized wound infection. Bacteriophages are released only in the presence of bacteria, significantly enhancing drug delivery efficiency and preventing indiscriminate medication use. This approach holds promise for extending therapeutic duration and reducing dosing frequency, representing a crucial step toward precision medicine [133]. The simulation prediction technique is a good approach for phage therapy research, as it provides a key support to ensure efficient and precise phage delivery to the target area by verifying phage release behavior through indirect experiments.

## 4. Limitations

Despite the remarkable progress in phage encapsulation technology, there are still several critical research gaps and challenges that need to be addressed before it can be widely applied in clinical and industrial settings.

### 4.1. Research on Encapsulation Technology

Alginate, chitosan, and liposomes provide good protection, but retention of phage infectivity after encapsulation tends to vary widely and lacks systematic quantification [134]. Further optimization of material composition and process parameters is needed in the future to minimize phage loss and extend shelf life. Moreover, the current research on the release rate is very lacking, which should be complemented in the study of encapsulation materials. Although encapsulation can enhance phage stability, achieving targeted delivery to specific infection sites remains an unsolved challenge. The development of smart controlled-release vectors capable of responding more precisely to environmental signals is still at an early stage and requires further in-depth research. The complexity of bacterial-phage interactions in the infection environment and the difficulty of simulating biofilms or polymicrobial environments in the laboratory lead to the fact that drug delivery strategies studied in vitro often fail in vivo. Researchers should conduct more in vivo experiments to ensure the usefulness of the results of research and development [134].

### 4.2. Scale Production

Current encapsulation technologies are mostly limited to the laboratory scale. It remains a challenge to translate these methods into a stable process that meets the requirements of industrial production and ensures consistency in particle size, encapsulation efficiency, and phage activity [135]. Problems such as batch-to-batch variation, inefficient encapsulation, and high production costs must be overcome through engineering innovation and process standardization. At the same time, production standards are difficult to control, and problems such as unclear Good Manufacturing Practice (GMP) guidelines, inconsistent endotoxin standards, and concentration limit contenders have slowed production development [136].

### 4.3. Security

The interaction between encapsulated phage and the host immune system is currently less studied [137]. Encapsulation may reduce immunogenicity by avoiding rapid phage clearance, but some materials may themselves cause an immune response [138]. In addition, there are potential risks associated with carrier accumulation or off-target release, such as the release of endotoxin from lysing Gram-negative bacteria, which may cause inflammatory reactions or even endotoxemia. This also needs to be systematically evaluated in relevant animal models and future clinical studies [138].

## 5. Discussion and Perspective

The emergence of phage therapy has significantly boosted the treatment of multidrug-resistant bacterial infections, providing a promising alternative to conventional antibiotics. However, the full potential of phage therapy has yet to be realized, particularly in terms of knowledge gaps in pharmacokinetics and pharmacodynamics (PK/PD), which have not kept pace with the escalating demand for this treatment modality [139]. Sustained research efforts are imperative to address these deficiencies and optimize treatment outcomes.

The development of phage cocktails requires the exploration of new phage combinations that minimize phage interferences, broaden the host range, and achieve precise and comprehensive bacterial killing. These advances are critical not only for clinical efficacy but also for applications such as food safety and environmental decontamination. In addition, phage engineering through CRISPR/Cas genome editing, homologous recombination, and synthetic phage DNA assembly needs to be explored more extensively. Engineered phage is the basis for the expansion of phage therapy and determines the upside of this therapy. Currently, there is limited research on CRISPR/Cas-based phage engineering, and increasing research in this area is critical to the potential of phage therapy. On the other hand, combination with other antimicrobial agents has also been found to potentially enhance the activity of phages. Research indicates that the combined use of bacteriophages and antibiotics has emerged as a novel strategy against biofilms. For instance, pretreatment with plasma-activated water effectively enhances the antibacterial activity of bacteriophages. In complex infection models such as wound biofilms and dual-bacterial communities, this combination therapy demonstrates synergistic effects, significantly improving overall antimicrobial efficacy [140,141,142].

The evolution of phage encapsulation technology clearly demonstrates a stepwise development path from fundamental optimization to functional integration. At the foundational level, traditional techniques such as those using alginate, chitosan nanoparticles, and liposomes were optimized primarily by adjusting material ratios and processes to enhance encapsulation efficiency and stability, laying the groundwork for subsequent applications. However, as the technology advances into the functional design phase for specific clinical applications, the strengths and weaknesses of different delivery routes become apparent: In oral delivery, physical particle encapsulation provides a robust barrier and excellent resistance to gastric acid, but its release mechanism is predominantly passive diffusion, lacking targeting and controllability [143]. In contrast, pH-responsive materials enable colon-targeted release, yet their insufficient mechanical strength may cause premature leakage in the stomach [144,145]. Furthermore, the similar pH levels in the small intestine and colon make precise control of the release site challenging. For wound healing, hydrogels are favored for their excellent biocompatibility and ability to maintain a moist environment, but their poor adhesion and inadequate mechanical strength may compromise efficacy. Electrospun fibers offer superior mechanical properties and controllable sustained release characteristics, yet their preparation is complex and costly [146]. For pulmonary delivery, dry powder inhalers (DPIs) excel at preserving phage activity and prolonging lung residence time, but drying stresses during preparation can inactivate phages; nebulized liquid formulations are simpler to prepare but suffer from poor stability and lung deposition efficiency [147,148]. These technical limitations have driven researchers to explore more advanced solutions. One promising direction involves developing composite encapsulation systems—such as combining chitosan nanoparticles with pH-responsive polymers, which may enhance system stability while enabling more precise targeted delivery—or integrating hydrogels with electrospun fibers to create novel dressings that combine excellent mechanical properties, biocompatibility, and stability. For dry powder inhalers, a “pre-encapsulation” strategy can be employed, where phages are first encapsulated in emulsions or liposomes before processing into dry powder, thereby providing effective protection during the drying process. Furthermore, cutting-edge research has advanced toward developing novel materials and intelligent responsive systems: innovative materials like spore wall capsules and green tea nanoparticles demonstrate the advantages of natural substances in providing unique protection and biocompatibility, while mimetic technologies have enabled a leap from in vitro validation to predictive design. This has ultimately led to the emergence of bio-responsive hydrogels capable of “on-demand release” triggered by bacterial activity itself. These advancements mark the transition of phage delivery from passive sustained release to an era of active, intelligent responsiveness. However, developing these composite materials is far from a simple accumulation of functions; it requires comprehensive consideration of the intricate interplay between material functionality, production processes, and cost. Future research should focus on deeper mechanistic analysis and efficacy validation of synergistic effects within these composite systems. By skillfully integrating the strengths of different materials and leveraging advanced simulation and prediction tools, the ultimate goal is to construct next-generation delivery systems capable of intelligently sensing and precisely responding to complex infection environments. This will propel phage therapeutics toward the critical leap from laboratory research to clinical application.

Future research should proactively shift toward technology pathways compatible with continuous production, such as utilizing microfluidic technology for precise control of nanoparticle synthesis or developing continuous electrospinning processes for efficient preparation of fiber dressings. When selecting materials and methods, it is imperative to establish a comprehensive evaluation system that transcends the singular metric of “encapsulation efficiency,” treating scalability, production costs, and batch-to-batch reproducibility as core indicators equally important to protective efficacy. Concurrently, the biosafety assessment of encapsulation systems must evolve from simple in vitro cytotoxicity testing toward more comprehensive immunopharmacological studies. This necessitates systematically analyzing the innate and adaptive immune responses potentially triggered by the carrier material itself during the preclinical phase. It also requires in-depth investigation into how encapsulation affects the distribution, metabolism, and clearance kinetics of phages within the body—data critical for predicting real immune responses in humans and avoiding potential inflammatory risks or carrier accumulation toxicity. Only by deeply integrating the design philosophy of “scalable production” with the research paradigm of “end-to-end immunological safety monitoring” can phage therapeutics be robustly advanced from innovative laboratory concepts to widely applied clinical products.

As these technological and safety hurdles are addressed, the scope of phage-based solutions is also expanding beyond their traditional antimicrobial role. Moreover, the potential of phage therapy extends far beyond treating human infectious diseases. Its high specificity and programmability are driving a range of interdisciplinary applications [149,150]. In human health, phages are being explored for precisely regulating the gut microbiome [151]. By targeting and eliminating specific pathogenic or opportunistic pathogens to restore ecological balance, they offer novel approaches for treating metabolic disorders, autoimmune diseases, and even neurological conditions [152]. In oncology, the excellent biocompatibility and modifiability of phage capsids make them ideal carriers for targeted delivery of anticancer drugs or imaging agents [153,154,155]. They can also be engineered for tumor diagnostic imaging and therapy [156]. In industrial and environmental applications, phage-based biofilm removal strategies are deployed as surface coatings for food processing pipelines, water treatment systems, and medical devices to prevent biofilm contamination, enhance safety, and extend service life [157]. In agriculture, phage preparations represent a green, precision biological control technology demonstrating significant potential in combating plant bacterial diseases caused by pathogens such as Xanthomonas and Erwinia [158,159,160]. This approach holds promise for reducing reliance on chemical pesticides, safeguarding food security, and promoting ecological sustainability. These emerging applications significantly expand the boundaries of phage technology, signaling its potential to play a pivotal role across multiple sectors.

Innovation: This review highlights composite material applications and explores a broader spectrum of delivery pathways, aiming to construct a more comprehensive knowledge framework for the reader. To strengthen the novelty of our work, we have incorporated a range of recent research advances to enrich the content. Furthermore, the article identifies current research gaps and challenges within phage technology, providing valuable insights and future directions for researchers.

## Figures and Tables

**Table 1:** Application of phage cocktails.

Types	Phages	Targets
multi-species phage cocktail	S19cd, S143_2, N2, C6, C2	*Salmonella*, *E. coli*
	Ph(S), Ph(E), Ph(P)	*Salmonella*, *E. coli*, *P. aeruginosa*
	VB_ECoM_SYGD1, VB ECoP SYGE1, VB ECOM SYGMH, vB_SauP_SLPW, vB SauM JDYN, vB_SauM JDF86, vB PaeS-PAJD-1	*E. coli*, *S. aureus*, *P. aeruginosa*
single-strain and single-species phage cocktail	JDYN, JDF86, SLPW	*S. aureus*
	vB_SauM_JDYN, vB_SauM_JDF86 and vB_SauP_SLPW	*S. aureus*
	HEMP1, HEMP4, HEMP5	*E. coli*

**Table 2:** Encapsulation materials to cope with different modes of delivery.

Delivery	Encapsulation Methods	Advantages	Disadvantages	Encapsulation Materials and Techniques	Target
Gastrointestinal	Particle encapsulation	Acid resistance Highly antibacterial Multifunctionality	Complex process Imprecise targeting	Cs-nps	*S. aureus*
	PH-responsive material	Acid resistance Precise targeting	Complex process Poor stability	Pectin	
				Methacrylic acid-ethyl acrylate copolymer	
				Alginate	
				Alginate-carrageenan	*Salmonella*
Wound	Hydrogel encapsulation	Biocompatibility Sustained effect	High cost Poor stability	Binary temperature-sensitive hydrogel consisting of Pf-127 and HPMC	*A. baumannii*
				Hydrogel consisting of sa, cmc and ha	*E.* *faecalis*
	Fiber encapsulation	Sustained effect Good stability	Complex process Material constraints	Electrospun fiber encapsulated materials	*P. aeruginosa*
Lung	Powder inhalation	Good stability long-term storage	Equipment requirements Complex process	Liposome encapsulation based on the thin-film hydration method	*P. aeruginosa*
				Inhalation pmps-pis	*S. aureus*
	Nebulized inhalation	Simple process High flexibility	Equipment requirements Poor stability Short shelf life	Liposome atomization encapsulation based on vibrating mesh nebulizer	
				Electrospun fiber encapsulated materials	*P. aeruginosa*

## Data Availability

No new data were created or analyzed in this study. Data sharing is not applicable to this article.

## References

[1] Zhao X., Van Overbeek L., Burgess C.M., Holden N., Brennan F., Johannessen G.S., Allende A., Höfte M., Cottyn B., Pothier J.F. (2025). Human Pathogenic Microorganisms in Fresh Produce Production: Lessons Learned When Plant Science Meets Food Safety. J. Food Prot..

[2] Chen B., Yang X., Yang C., Chan E.W.-C., Wang X., Chen S. (2025). Ultrasensitive monoclonal antibody-based Salmonella detection through fluorescence single bacterial cell imaging selectively enhanced by tyramide signal amplification. Innov. Food Sci. Emerg. Technol..

[3] Sripaurya B., Pelyuntha W., Ngasaman R., Ching C.L., Guyonnet V., Vongkamjan K. (2025). Antibiotic resistance, sequence typing, and virulence gene profiles of Salmonella enterica isolated from the broiler production chain in southern Thailand. Res. Vet. Sci..

[4] Liu Z., Liu Y., He J., Pei X., Lin Y., Magnani M., Liu D., Ding T., Feng J. (2025). Milkfat influences thermal tolerance and biofilm formation of Salmonella Typhimurium during pasteurization. Food Res. Int..

[5] Polat İ., Güngör İ., Şen B. (2025). Prevalence of Salmonella enterica Serotypes Isolated From Broiler Liver and Their Antibiotic Resistance Profiles. J. Food Prot..

[6] Khanal S., Luitel H., Adhikari S., Marasini A., Bhattarai R.K. (2025). Molecular characterization, antimicrobial resistance profiling, and biofilm analysis of Salmonella isolates from dead-in-shell embryonated eggs. Poult. Sci..

[7] Woh P.Y., Chen Y., Nurjadi D. (2025). Bridging the phylodynamic antimicrobial resistance of Salmonella in Southeast Asia and Hong Kong through a One Health Lens. Int. J. Food Microbiol..

[8] Zhan Z., Zhou Z., Hu M., Tai C., Shi X. (2025). Characterization of colistin resistance mechanisms development in Salmonella isolates from retail meat in Shanghai, China. LWT.

[9] Xu J., Wang J., Kang Z., Xu H., Tang C., Chen J., Hu H. (2025). Mixed-species biofilm with Salmonella in food industry: Persistence, interspecies interaction, and control. Food Res. Int..

[10] Thiers I., Lissens M., Langie H., Lories B., Steenackers H. (2024). Salmonella biofilm formation diminishes bacterial proliferation in the C. elegans intestine. Biofilm.

[11] Tulin G., Méndez A.A.E., Figueroa N.R., Smith C., Folmer M.P., Serra D., Wade J.T., Checa S.K., Soncini F.C. (2025). Integration of BrfS into the biofilm-controlling cascade promotes sessile Salmonella growth at low temperatures. Biofilm.

[12] Liang T., Liang Z., Wu S., Ding Y., Wu Q., Gu B. (2023). Global prevalence of Staphylococcus aureus in food products and its relationship with the occurrence and development of diabetes mellitus. Med. Adv..

[13] Apiratwarrasakul S., Sresuwadjarey P., Phumthanakorn N., Withatanung P., Thongdee M., Lerdsittikul V. (2025). The broad-spectrum kayvirus phage disrupts biofilms formed by methicillin-resistant Staphylococcus aureus and methicillin-resistant Staphylococcus pseudintermedius. Virology.

[14] Konatam S., Roy D.N. (2025). GC-MS unveils monosaccharide D-Allose in Terminalia arjuna bark extract acting against biofilm regulatory proteins (SrtA and SarA) of Staphylococcus aureus: A drug discovery approach. Int. J. Mass Spectrom..

[15] Rana E.A., Nur S., Fazal M.A., Alam M., Ahasan A.S.M.L., Rocky M.J.H., Rakib T.M. (2025). Genome sequences of three methicillin-resistant Staphylococcus aureus strains isolated from subclinical mastitic cows. Microbiol. Resour. Announc..

[16] Cheung G.Y.C., Bae J.S., Otto M. (2021). Pathogenicity and virulence of Staphylococcus aureus. Virulence.

[17] Ahmad-Mansour N., Loubet P., Pouget C., Dunyach-Remy C., Sotto A., Lavigne J.P., Molle V. (2021). Staphylococcus aureus Toxins: An Update on Their Pathogenic Properties and Potential Treatments. Toxins.

[18] Sivori F., Cavallo I., Truglio M., De Maio F., Sanguinetti M., Fabrizio G., Licursi V., Francalancia M., Fraticelli F., La Greca I. (2024). Staphylococcus aureus colonizing the skin microbiota of adults with severe atopic dermatitis exhibits genomic diversity and convergence in biofilm traits. Biofilm.

[19] Turner A.B., Zermeño-Pérez D., Mysior M.M., Giraldo-Osorno P.M., García B., O’Gorman E., Oubihi S., Simpson J.C., Lasa I., Cróinín T.Ó (2024). Biofilm morphology and antibiotic susceptibility of methicillin-resistant *Staphylococcus aureus* (MRSA) on poly-D,L-lactide-co-poly(ethylene glycol) (PDLLA-PEG) coated titanium. Biofilm.

[20] Rahal E.A., Kazzi N., Nassar F.J., Matar G. (2012). Escherichia coli O157: H7—Clinical aspects and novel treatment approaches. Front. Cell. Infect. Microbiol..

[21] Rangel J.M., Sparling P.H., Crowe C., Griffin P.M., Swerdlow D. (2005). Epidemiology of Escherichia coli O157: H7 outbreaks, united states, 1982–2002. Emerg. Infect. Dis..

[22] Charron R., Lemée P., Huguet A., Minlong O., Boulanger M., Houée P., Soumet C., Briandet R., Bridier A. (2025). Strain-dependent emergence of aminoglycoside resistance in Escherichia coli biofilms. Biofilm.

[23] Zhang T., Ray S., Melican K., Richter-Dahlfors A. (2024). The maturation of native uropathogenic Escherichia coli biofilms seen through a non-interventional lens. Biofilm.

[24] Lin Z., Duan X., Wan X., Han S., Ji L., Zeng Y., Shen J., Lu H. (2025). Coevolutionary phage training expands phage host range: Driven by tail fiber mutations in Pseudomonas aeruginosa lytic phage?. Microbiol. Res..

[25] Horcajada J.P., Gales A., Isler B., Kaye K.S., Kwa A.L., Landersdorfer C.B., Montero M.M., Oliver A., Pogue J.M., Shields R.K. (2025). How do I manage difficult-to-treat Pseudomonas aeruginosa infections? Key questions for today’s clinicians. Clin. Microbiol. Infect..

[26] Alvarez C., Bello J., Vay C., Famiglietti Á., Nastro M., Rodríguez H. Antimicrobial resistance profiles in multidrug-resistant Pseudomonas aeruginosa isolates in a university hospital in Argentina. Activity of new antibiotic combinations. Rev. Argent. Microbiol..

[27] Tuon F.F., Dantas L.R., Suss P.H., Tasca Ribeiro V.S. (2022). Pathogenesis of the Pseudomonas aeruginosa Biofilm: A Review. Pathogens.

[28] Silva B.S., Furtado M.M., Freire L., Lee S., Jorge G.P., Daldosso G., Silva C.E., Vieira-Junior L., Lemos Junior W.J.F., Oliveira C.A.F. (2025). A large sampling study on the occurrence and characteristics of Pseudomonas aeruginosa and heterotrophic bacteria in mineral water over seasons and in different containers. Int. J. Food Microbiol..

[29] Fernández-Billón M., Llambías-Cabot A.E., Jordana-Lluch E., Oliver A., Macià M.D. (2023). Mechanisms of antibiotic resistance in Pseudomonas aeruginosa biofilms. Biofilm.

[30] Glen K.A., Lamont I.L. (2021). β-lactam Resistance in Pseudomonas aeruginosa: Current Status, Future Prospects. Pathogens.

[31] Schlichter Kadosh Y., Muthuraman S., Nisaa K., Ben-Zvi A., Karsagi Byron D.L., Shagan M., Brandis A., Mehlman T., Gopas J., Saravana Kumar R. (2024). Pseudomonas aeruginosa quorum sensing and biofilm attenuation by a di-hydroxy derivative of piperlongumine (PL-18). Biofilm.

[32] Rima M., Villeneuve-Faure C., Pilloux L., Roques C., El Garah F., Makasheva K. (2025). From adhesion to biofilms formation and resilience: Exploring the impact of silver nanoparticles-based biomaterials on Pseudomonas aeruginosa. Biofilm.

[33] Steinle J.S., Bertolini A.B., Pereira J.G., Paes A.C., da Silva L.P., dos Santos Leite B., Ribeiro T.M., Aires I.N., Rangel M.V., Curci V.C.M. (2025). Molecular detection of Listeria monocytogenes in refrigerated raw milk and geospatial distribution in Brazilian small farms. Food Sci. Technol..

[34] Fan C., Wang Y., Zheng T., Li W., Li X., Wu P. (2025). Rapid detection of Listeria monocytogenes in ready-to-eat foods using a one-tube recombinase polymerase amplification and photosensitization colorimetric assay. Sens. Actuators B Chem..

[35] França L., Rauecker U.N., Santos D.L.S.d., Almeida N.A., Gebara C., Prado C.S., Cavicchioli V.Q., Lage M.E., Duarte F.O., Rezende C.S.M.E. (2025). Occurrence of *Listeria* spp. and *Listeria monocytogenes* in half-carcasses, meat cuts, equipment, and the environment of bovine slaughterhouses in Brazil. Res. Vet. Sci..

[36] Li Z., Shao Y., Liu X., Wan X., Xiong P., Wang L., Yuan J. (2025). Steap3 is a key node in regulating the phagosome escape of Listeria monocytogenes. Mol. Immunol..

[37] Upham J.P., Markell J.A. (2025). Challenge Testing to Determine Growth of Listeria monocytogenes on Fresh Enoki Mushrooms at 4 °C and 10 °C Storage Temperatures. J. Food Prot..

[38] Bechtel T.D., Hershelman J., Ghoshal M., McLandsborough L., Gibbons J. (2024). Chemical mutagenesis of Listeria monocytogenes for increased tolerance to benzalkonium chloride shows independent genetic underpinnings and off-target antibiotic resistance. PLoS ONE.

[39] Rolon M.L., Voloshchuk O., Bartlett K.V., LaBorde L.F., Kovac J. (2024). Multi-species biofilms of environmental microbiota isolated from fruit packing facilities promoted tolerance of Listeria monocytogenes to benzalkonium chloride. Biofilm.

[40] Liu X., Liu S., Wang Y., Shi Y., Chen Q. (2024). New insights into the antibiofilm activity and mechanism of Mannosylerythritol Lipid-A against Listeria monocytogenes EGD-e. Biofilm.

[41] Tang K.W.K., Millar B.C., Moore J.E. (2023). Antimicrobial resistance (AMR). Br. J. Biomed. Sci..

[42] Asmare Z., Tamrat E., Erkihun M., Endalamaw K., Alelign D., Getie M., Sisay A., Gashaw Y., Reta M.A. (2025). Antimicrobial resistance pattern of Acinetobacter baumannii clinical isolate in Ethiopia. A systematic review and meta-analysis. Syst. Rev. Meta-Anal..

[43] Antochevis L.C., Wilhelm C.M., Arns B., Sganzerla D., Sudbrack L.O., Nogueira T.C., Guzman R.D., Martins A.S., Cappa D.S., Dos Santos Â.C. (2025). World Health Organization priority antimicrobial resistance in Enterobacterales, Acinetobacter baumannii, Pseudomonas aeruginosa, Staphylococcus aureus and Enterococcus faecium healthcare-associated bloodstream infections in Brazil (ASCENSION): A prospective, multicentre, observational study. Lancet Reg. Health–Am..

[44] Pallett S.J., Morkowska A., Woolley S.D., Potochilova V.V., Rudnieva K.L., Iungin O.S., Sgro V., Boyd S.E., Reece N., Lambert Z.L. (2025). Evolving antimicrobial resistance of extensively drug-resistant Gram-negative severe infections associated with conflict wounds in Ukraine: An observational study. Lancet Reg. Health–Eur..

[45] Yin C., Alam M.Z., Fallon J.T., Huang W. (2024). Advances in development of novel therapeutic strategies against multi-drug resistant Pseudomonas aeruginosa. Antibiotics.

[46] Naghavi M., Vollset S.E., Ikuta K.S., Swetschinski L.R., Gray A.P., Wool E.E., Aguilar G.R., Mestrovic T., Smith G., Han C. (2024). Global burden of bacterial antimicrobial resistance 1990–2021: A systematic analysis with forecasts to 2050. Arch. Virol..

[47] Villalpando-Aguilar J.L., Matos-Pech G., López-Rosas I., Castelán-Sánchez H.G., Alatorre-Cobos F. (2023). Phage therapy for crops: Concepts, experimental and bioinformatics approaches to direct Its application. Int. J. Mol. Sci..

[48] Ackermann H.-W. (2007). 5500 Phages examined in the electron microscope. Arch. Virol..

[49] Strathdee S.A., Hatfull G.F., Mutalik V.K., Schooley R.T. (2023). Phage therapy: From biological mechanisms to future directions. Cell.

[50] Munteanu D.I., Dunn J., Apjok G., Kintses B., Griselain J., Steurs G., Cochez C., Djebara S., Merabishvili M., Pirnay J.P. (2025). Phage Therapy for Orthopaedic Infections: The First Three Cases from the United Kingdom. Antibiotics.

[51] Teklemariam A.D., Al-Hindi R.R., Qadri I., Alharbi M.G., Ramadan W.S., Ayubu J., Al-Hejin A.M., Hakim R.F., Hakim F.F., Hakim R.F. (2023). The Battle between Bacteria and Bacteriophages: A Conundrum to Their Immune System. Antibiotics.

[52] Helmy Y.A., Taha-Abdelaziz K., Hawwas H.A.E.-H., Ghosh S., AlKafaas S.S., Moawad M.M., Saied E.M., Kassem I.I., Mawad A. (2023). Antimicrobial resistance and recent alternatives to antibiotics for the control of bacterial pathogens with an emphasis on foodborne pathogens. Antibiotics.

[53] Taj M.I., Ding Y., Bao Y., Huang Y., Guan P., Zhang Y., Liu X., Ming Z., Wang X. (2024). Biocontrol characteristics and application of phage SEP4 against multidrug-resistant Salmonella biofilm on food matrix. Food Biosci..

[54] Teklemariam A.D., Al Hindi R., Qadri I., Alharbi M.G., Hashem A.M., Alrefaei A.A., Basamad N.A., Haque S., Alamri T., Harakeh S. (2024). Phage cocktails–an emerging approach for the control of bacterial infection with major emphasis on foodborne pathogens. Biotechnol. Genet. Eng. Rev..

[55] Pelyuntha W., Ngasaman R., Yingkajorn M., Chukiatsiri K., Guyonnet V., Vongkamjan K. (2024). Phage cocktail administration to reduce Salmonella load in broilers. Res. Vet. Sci..

[56] Shin H., Ahn E., Kim J., Ryu S. (2024). Development of phage cocktail for controlling colistin-resistant Escherichia coli harboring mcr-1 and its biofilm formation in retail raw chicken. LWT.

[57] Guo M., Zhang Y., Wu L., Xiong Y., Xia L., Cheng Y., Ma J., Wang H., Sun J., Wang Z. (2024). Development and mouse model evaluation of a new phage cocktail intended as an alternative to antibiotics for treatment of Staphylococcus aureus-induced bovine mastitis. J. Dairy Sci..

[58] Liu Y., Lin Y., Zhu W. (2024). Systemic effects of a phage cocktail on healthy weaned piglets. Biology.

[59] Shiue S.J., Wu M.S., Chiang Y.H., Lin H.Y. (2024). Bacteriophage—Cocktail hydrogel dressing to prevent multiple bacterial infections and heal diabetic ulcers in mice. J. Biomed. Mater. Res. Part A.

[60] Xue Y., Gao Y., Guo M., Zhang Y., Zhao G., Xia L., Ma J., Cheng Y., Wang H., Sun J. (2024). Phage cocktail superimposed disinfection: A ecological strategy for preventing pathogenic bacterial infections in dairy farms. Environ. Res..

[61] Królikowska D., Szymańska M., Krzyżaniak M., Guziński A., Matusiak R., Kajdanek A., Kaczorek-Łukowska E., Maszewska A., Wójcik E.A., Dastych J. (2024). A New Approach for Phage Cocktail Design in the Example of Anti-mastitis Solution. Pathogens.

[62] Hegarty B. (2025). Making waves: Intelligent phage cocktail design, a pathway to precise microbial control in water systems. Water Res..

[63] Kunisch F., Campobasso C., Wagemans J., Yildirim S., Chan B.K., Schaudinn C., Lavigne R., Turner P.E., Raschke M.J., Trampuz A. (2024). Targeting Pseudomonas aeruginosa biofilm with an evolutionary trained bacteriophage cocktail exploiting phage resistance trade-offs. Nat. Commun..

[64] Li C., Shi T., Sun Y., Zhang Y. (2022). A novel method to create efficient phage cocktails via use of phage-resistant bacteria. Appl. Environ. Microbiol..

[65] Yuan X., Fan L., Jin H., Wu Q., Ding Y. (2025). Phage engineering using synthetic biology and artificial intelligence to enhance phage applications in food industry. Curr. Opin. Food Sci..

[66] Mahony J. (2024). Biological and bioinformatic tools for the discovery of unknown phage–host combinations. Curr. Opin. Microbiol..

[67] Keith M., Park de la Torriente A., Chalka A., Vallejo-Trujillo A., McAteer S.P., Paterson G.K., Low A.S., Gally D.L. (2024). Predictive phage therapy for Escherichia coli urinary tract infections: Cocktail selection for therapy based on machine learning models. Proc. Natl. Acad. Sci. USA.

[68] Lood C., Boeckaerts D., Stock M., De Baets B., Lavigne R., van Noort V., Briers Y.J.C.O.i.V. (2022). Digital phagograms: Predicting phage infectivity through a multilayer machine learning approach. Curr. Opin. Virol..

[69] Smug B.J., Majkowska-Skrobek G., Drulis-Kawa Z. (2022). PhREEPred: Phage Resistance Emergence Prediction Web Tool to Foresee Encapsulated Bacterial Escape from Phage Cocktail Treatment. J. Mol. Biol..

[70] Seguritan V., Alves Jr N., Arnoult M., Raymond A., Lorimer D., Burgin A.B., Salamon P., Segall A.M. (2012). Artificial neural networks trained to detect viral and phage structural proteins. PLoS Comput. Biol..

[71] Peng H., Chen I.A., Qimron U. (2024). Engineering phages to fight multidrug-resistant bacteria. Chem. Rev..

[72] Nidhi S., Anand U., Oleksak P., Tripathi P., Lal J.A., Thomas G., Kuca K., Tripathi V. (2021). Novel CRISPR–Cas systems: An updated review of the current achievements, applications, and future research perspectives. Int. J. Mol. Sci..

[73] Stoltzfus M.J., Workman R.E., Keith N.C., Modell J.W. (2024). A dynamic subpopulation of CRISPR–Cas overexpressers allows Streptococcus pyogenes to rapidly respond to phage. Nat. Microbiol..

[74] Workman R.E., Stoltzfus M.J., Keith N.C., Euler C.W., Bondy-Denomy J., Modell J.W. (2024). Anti-CRISPR proteins trigger a burst of CRISPR-Cas9 expression that enhances phage defense. Cell Rep..

[75] Koonin E.V., Makarova K.S., Zhang F. (2017). Diversity, classification and evolution of CRISPR-Cas systems. Curr. Opin. Microbiol..

[76] Ran F.A., Hsu P.D., Wright J., Agarwala V., Scott D.A., Zhang F. (2013). Genome engineering using the CRISPR-Cas9 system. Nat. Protoc..

[77] Jia H.-J., Jia P.-P., Yin S., Bu L.-K., Yang G., Pei D.-S. (2023). Engineering bacteriophages for enhanced host range and efficacy: Insights from bacteriophage-bacteria interactions. Front. Microbiol..

[78] Hidalgo-Cantabrana C., Barrangou R. (2020). Characterization and applications of Type I CRISPR-Cas systems. Biochem. Soc. Trans..

[79] Gencay Y.E., Jasinskytė D., Robert C., Semsey S., Martínez V., Petersen A.Ø., Brunner K., de Santiago Torio A., Salazar A., Turcu I.C. (2024). Engineered phage with antibacterial CRISPR–Cas selectively reduce E. coli burden in mice. Nat. Biotechnol..

[80] Chylinski K., Makarova K.S., Charpentier E., Koonin E.V. (2014). Classification and evolution of type II CRISPR-Cas systems. Nucleic Acids Res..

[81] Agha A.S.A., Al-Samydai A., Aburjai T. (2025). New frontiers in CRISPR: Addressing antimicrobial resistance with Cas9, Cas12, Cas13, and Cas14. Heliyon.

[82] Hosen A., Nishat M.N.H., Soaib M.M.H., Sharkar O.S., Sahabuddin M., Sharif I.H., Bhajan S.K. (2025). A Review: CRISPR Cas System and the Mechanism With an Inhibition of Binding of CRISPR Cas—9. Nano Sel..

[83] Tsubota T., Takasu Y., Yonemura N., Sezutsu H. (2025). Enhancements of the CRISPR-Cas System in the Silkworm Bombyx mori. Cris. J..

[84] Park J.Y., Moon B.Y., Park J.W., Thornton J.A., Park Y.H., Seo K.S. (2017). Genetic engineering of a temperate phage-based delivery system for CRISPR/Cas9 antimicrobials against Staphylococcus aureus. Sci. Rep..

[85] Ranveer S.A., Dasriya V., Ahmad M.F., Dhillon H.S., Samtiya M., Shama E., Anand T., Dhewa T., Chaudhary V., Chaudhary P. (2024). Positive and negative aspects of bacteriophages and their immense role in the food chain. NPJ Sci. Food.

[86] Wagh R.V., Priyadarshi R., Rhim J.-W. (2023). Novel bacteriophage-based food packaging: An innovative food safety approach. Coatings.

[87] Colom J., Cano-Sarabia M., Otero J., Cortés P., Maspoch D., Llagostera M. (2015). Liposome-encapsulated bacteriophages for enhanced oral phage therapy against *Salmonella* spp.. Appl. Environ. Microbiol..

[88] Yang Y., Du H., Zou G., Song Z., Zhou Y., Li H., Tan C., Chen H., Fischetti V.A., Li J. (2023). Encapsulation and delivery of phage as a novel method for gut flora manipulation in situ: A review. J. Control. Release.

[89] Koçer Alaşalvar G., Yıldırım Z.J. (2024). Encapsulation of SE—P47 phage specific to Salmonella Enteritidis and evaluation of its stability. J. Food Saf..

[90] Selim H.M.R.M., Gomaa F.A.M., Alshahrani M.Y., Morgan R.N., Aboshanab K.M. (2025). Phage therapeutic delivery methods and clinical trials for combating clinically relevant pathogens. Ther. Deliv..

[91] Choi I., Lee J.-S., Han J. (2023). Maltodextrin-trehalose miscible system-based bacteriophage encapsulation: Studies of plasticizing effect on encapsulated phage activity and food application as an antimicrobial agent. Food Control.

[92] Guo P., Zhang L., Ning M., Cai T., Long F., Yuan Y., Yue T. (2025). Characterization and release of casein—Sodium alginate embedding phage edible film and the application in controlling of Salmonella contamination in food. Int. J. Food Microbiol..

[93] Simadibrata D.M., Syam A.F., Lee Y.Y. (2022). A comparison of efficacy and safety of potassium—Competitive acid blocker and proton pump inhibitor in gastric acid—Related diseases: A systematic review and meta—Analysis. J. Gastroenterol. Hepatol..

[94] Javeedvali S., Gopalakrishnan C., Kannan R., Manonmani S., Prasanthrajan M., Varanavasiappan S. (2024). Strategies for sustainable rice bacterial leaf blight management: A holistic approach through phage biocontrol and nanoparticle encapsulation. J. Plant Pathol..

[95] Xu Y., Yang T., Miao Y., Zhang Q., Yang M., Mao C. (2024). Injectable Phage-Loaded Microparticles Effectively Release Phages to Kill Methicillin-Resistant Staphylococcus aureus. ACS Appl. Mater. Interfaces.

[96] Elsayed M.M., Elkenany R.M., El-Khateeb A.Y., Nabil N.M., Tawakol M.M., Hassan H.M. (2024). Isolation and encapsulation of bacteriophage with chitosan nanoparticles for biocontrol of multidrug-resistant methicillin-resistant Staphylococcus aureus isolated from broiler poultry farms. Sci. Rep..

[97] Temsaah H.R., Abdelkader K., Ahmed A.E., Elgiddawy N., Eldin Z.E., Elshebrawy H.A., Kasem N.G., El-Gohary F.A., Azmy A.F. (2025). Chitosan nano-formulation enhances stability and bactericidal activity of the lytic phage HK6. BMC Biotechnol..

[98] Karthika C., Malligarjunan N., Pandian S.K., Gowrishankar S. (2025). Chitosan-encapsulated bacteriophage cocktail as promising oral delivery system to surpass gastrointestinal infection caused by Klebsiella aerogenes. Int. J. Biol. Macromol..

[99] Salatin S., Yari Khosroushahi A. (2017). Overviews on the cellular uptake mechanism of polysaccharide colloidal nanoparticles. J. Cell Mol. Med..

[100] Li J., Ma L., Wang C., Jiang P., Cui P., Wang J. (2022). Rationally designed oral DOX gels for colon-specific administration. Gels.

[101] Guan Z., Feng Q. (2022). Chitosan and chitooligosaccharide: The promising non-plant-derived prebiotics with multiple biological activities. Int. J. Mol. Sci..

[102] Li Y., Li X.-M., Duan H.-Y., Yang K.-D., Ye J.-F. (2024). Advances and optimization strategies in bacteriophage therapy for treating inflammatory bowel disease. Front. Immunol..

[103] Lin S., Xie G., He J., Meng L., Pang Y., Liu J. (2025). Enhancing phage therapy by coating single bacteriophage-infected bacteria with polymer to preserve phage vitality. Nat. Biomed. Eng..

[104] Chen H., Zhi W., Bai B., Anjum F.R., Jia Z., Kong R., Liu Q., Wang B., Ma C., Ma D. (2025). Impact of sodium alginate hydrogel containing bacteriophage peptides that specifically bind to the EtCab protein on the inhibition of Eimeria tenella infection. Vet. Res..

[105] Dlamini S.B., Gigante A.M., Hooton S.P., Atterbury R.J. (2023). Efficacy of different encapsulation techniques on the viability and stability of diverse phage under simulated gastric conditions. Microorganisms.

[106] Truong-Le Q.A., Lee S.O., Ubeyitogullari A. (2025). Encapsulation of Bifidobacterium bifidum into a pH-sensitive alginate-pectin gel system using 3D food printing: Enhanced viability and targeted release. Int. J. Biol. Macromol..

[107] Guadarrama-Escobar O.R., Sánchez-Vázquez I., Serrano-Castañeda P., Chamorro-Cevallos G.A., Rodríguez-Cruz I.M., Sánchez-Padrón A.Y., Anguiano-Almazán E., Peña-Juárez M.C., Méndez-Albores A., Domínguez-Delgado C.L. (2021). Development, Characterization, Optimization, and In Vivo Evaluation of Methacrylic Acid-Ethyl Acrylate Copolymer Nanoparticles Loaded with Glibenclamide in Diabetic Rats for Oral Administration. Pharmaceutics.

[108] Li N., Zhang G., Zhang X., Liu Y., Kong Y., Wang M., Ren X. (2024). A rapid-floating natural polysaccharide gel-raft with double-effect for the treatment of gastroesophageal reflux disease. Int. J. Biol. Macromol..

[109] Brinck J.E., Sinha A.K., Laursen M.F., Dragsted L.O., Raes J., Uribe R.V., Walter J., Roager H.M., Licht T.R. (2025). Intestinal pH: A major driver of human gut microbiota composition and metabolism. Nat. Rev. Gastroenterol. Hepatol..

[110] Mukhopadhyay S., To K.K., Liu Y., Bai C., Leung S.S. (2024). A thermosensitive hydrogel formulation of phage and colistin combination for the management of multidrug-resistant Acinetobacter baumannii wound infections. Biomater. Sci..

[111] Abed S., Beig M., Barzi S.M., Shafiei M., Hashemi Shahraki A., Sadeghi S., Sohrabi A. (2024). Development of phage-containing hydrogel for treating Enterococcus faecalis-infected wounds. PLoS ONE.

[112] Khazani Asforooshani M., Elikaei A., Abed S., Shafiei M., Barzi S.M., Solgi H., Badmasti F., Sohrabi A. (2024). A novel Enterococcus faecium phage EF-M80: Unveiling the effects of hydrogel-encapsulated phage on wound infection healing. Front. Microbiol..

[113] Peng X., Wang T., Dai B., Zhu Y., Ji M., Yang P., Zhang J., Liu W., Miao Y., Liu Y. (2025). Gene Therapy for Inflammatory Cascade in Intrauterine Injury with Engineered Extracellular Vesicles Hybrid Snail Mucus-enhanced Adhesive Hydrogels. Adv. Sci..

[114] An D., Wang Z., Ning Y., Yue Y., Xuan H., Hu Y., Yang M., Zhou H., Liu Q., Wang X. (2024). One-Step Physical and Chemical Dual-Reinforcement with Hydrophobic Drug Delivery in Gelatin Hydrogels for Antibacterial Wound Healing. ACS Omega.

[115] Shakeri A., Najm L., Khan S., Tian L., Ladouceur L., Sidhu H., Al-Jabouri N., Hosseinidoust Z., Didar T.F. (2024). Noncontact 3D Bioprinting of Proteinaceous Microarrays for Highly Sensitive Immunofluorescence Detection within Clinical Samples. ACS Nano.

[116] Dehnad D., Emadzadeh B., Ghorani B., Rajabzadeh G., Tucker N., Jafari S.M. (2023). Bioactive-loaded nanovesicles embedded within electrospun plant protein nanofibers; a double encapsulation technique. Food Hydrocoll..

[117] Xu C., Ma J., Liu Z., Wang W., Liu X., Qian S., Chen L., Gu L., Sun C., Hou J. (2023). Preparation of shell-core fiber-encapsulated Lactobacillus rhamnosus 1.0320 using coaxial electrospinning. Food Chem..

[118] Ju T., Li J., Weston A., Satta G., Bolognini S., Di Luca M., Gaisford S., Williams G.R. (2025). Anti—Pseudomonas Aeruginosa Bacteriophage Loaded Electrospun Fibers for Antibacterial Wound Dressings. Macromol. Rapid Commun..

[119] Murray C.J., Ikuta K.S., Sharara F., Swetschinski L., Aguilar G.R., Gray A., Han C., Bisignano C., Rao P., Wool E. (2022). Global burden of bacterial antimicrobial resistance in 2019: A systematic analysis. Lancet.

[120] Khosravi A., Chen Q., Echterhof A., Koff J.L., Bollyky P.L. (2024). Phage therapy for respiratory infections: Opportunities and challenges. Lung.

[121] Baldelli A., Liang M. (2023). Design of respirable sprayed microparticles of encapsulated bacteriophages. Front. Drug Deliv..

[122] Sawant S.S., Ahmed M.U., Gantala N.-G., Chiu C., Qu L., Zhou Q. (2025). Development of Inhalable Bacteriophage Liposomes Against Pseudomonas aeruginosa. Pharmaceutics.

[123] Liu M.-Y., Liu X., Wang C.-Y., Wan Q.-Q., Tian Y.-F., Liu S.-L., Pang D.-W., Wang Z.-G. (2024). Inhalable polymeric microparticles for phage and photothermal synergistic therapy of Methicillin-Resistant Staphylococcus aureus pneumonia. Nano Lett..

[124] Li J., Zheng H., Leung S.S.Y. (2024). Investigating the effectiveness of liposome-bacteriophage nanocomplex in killing Staphylococcus aureus using epithelial cell coculture models. Int. J. Pharm..

[125] Cao Y., Wang Y., Li M., Khanal D., Chan H.K. (2025). Can bacteriophage be stabilised by lipid encapsulation when nebulised for inhalation delivery against Pseudomonas aeruginosa?. Int. J. Pharm..

[126] Liu S., Quek S.Y., Huang K. (2024). An Ecofriendly Nature—Inspired Microcarrier for Enhancing Delivery, Stability, and Biocidal Efficacy of Phage—Based Biopesticides. Small.

[127] Yaacob S., Jamil R.Z.R., Suah F.B.M. (2022). Sporopollenin based materials as a versatile choice for the detoxification of environmental pollutants—A review. Int. J. Biol. Macromol..

[128] Singh L.S. (2024). Nano-emulsion encapsulation for the efficient delivery of bacteriophage therapeutics. Biologicals.

[129] Wdowiak M., Raza S., Grotek M., Zbonikowski R., Nowakowska J., Doligalska M., Cai N., Luo Z., Paczesny J. (2025). Phage-Nanoparticle Cocktails as a Novel Antibacterial Approach: Synergistic Effects of Bacteriophages and Green-Synthesized Silver Nanoparticles. BioRxiv.

[130] Easwaran M., Raja N., Saravanan M., Belete M.A. (2024). Liposome-loaded phage cocktail: A promising therapeutic option against postsurgical wound infections–A critical appraisal. Ann. Med. Surg..

[131] Zhang B., Wang Y., Wang F., Zhang Y., Hao H., Lv X., Hao L., Shi Y. (2022). Microencapsulated phage composites with increased gastrointestinal stability for the oral treatment of Salmonella colonization in chicken. Front. Vet. Sci..

[132] Jamaledin R., Sartorius R., Di Natale C., Onesto V., Manco R., Mollo V., Vecchione R., De Berardinis P., Netti P.A. (2023). PLGA microparticle formulations for tunable delivery of a nano-engineered filamentous bacteriophage-based vaccine: In vitro and in silico-supported approach. J. Nanostruct. Chem..

[133] Tao S., Hu A., Bavel E., Yu C.H., Zhang S., Kissling V.M., Li Z., Moriaty T.F., Maniura-Weber K., Ren Q. (2025). Bio—Responsive Hydrogel for Targeted on—Demand Release of a Phage Cocktail for Treatment of Pseudomonas aeruginosa Infection. Adv. Funct. Mater..

[134] Youssef R.A., Sakr M.M., Shebl R.I., Aboshanab K.M. (2025). Recent insights on challenges encountered with phage therapy against gastrointestinal-associated infections. Gut Pathog..

[135] Taati Moghadam M., Mohebi S., Sheikhi R., Hasannejad-Bibalan M., Shahbazi S., Nemati S. (2025). Phage and Endolysin Therapy Against Antibiotics Resistant Bacteria: From Bench to Bedside. MedComm.

[136] Mohammadi E., Rahimian M., Panahi B. (2025). Bridging the gap: Phage manufacturing processes from laboratory to agri-food industry. Virus Res..

[137] Fatima R., Hynes A.P. (2025). Phage-Antibiotic Combinations for Pseudomonas: Successes in the Clinic and In Vitro Tenuously Connected. Microb. Biotechnol..

[138] Banicod R.J.S., Javaid A., Tabassum N., Jo D.M., Hassan M.I., Kim Y.M., Khan F. (2025). Marine Bacteriophages as Next-Generation Therapeutics: Insights into Antimicrobial Potential and Application. Viruses.

[139] Nang S.C., Lin Y.-W., Fabijan A.P., Chang R.Y., Rao G.G., Iredell J., Chan H.-K., Li J. (2023). Pharmacokinetics/pharmacodynamics of phage therapy: A major hurdle to clinical translation. Clin. Microbiol. Infect..

[140] Akturk E., Pinto G., Ostyn L., Crabbé A., Melo L.D.R., Azeredo J., Coenye T. (2025). Combination of phages and antibiotics with enhanced killing efficacy against dual-species bacterial communities in a three-dimensional lung epithelial model. Biofilm.

[141] Akturk E., Melo L.D.R., Oliveira H., Crabbé A., Coenye T., Azeredo J. (2023). Combining phages and antibiotic to enhance antibiofilm efficacy against an in vitro dual species wound biofilm. Biofilm.

[142] Shambharkar A., Thompson T.P., McClenaghan L.A., Bourke P., Gilmore B.F., Skvortsov T. (2024). Plasma activated water pre-treatment substantially enhances phage activity against Proteus mirabilis biofilms. Biofilm.

[143] Sokolov I. (2024). Ultrabright fluorescent particles via physical encapsulation of fluorescent dyes in mesoporous silica: A mini-review. Nanoscale.

[144] Zhang X., Liu Y., Wang Z., Zhao H., Zhan L., Gui H., Xu X., Ma X., Ma B. (2025). pH-responsive and self-adaptive injectable sodium alginate/carboxymethyl chitosan hydrogel accelerates infected wound healing by bacteriostasis and immunomodulation. Carbohydr. Polym..

[145] Xue J., Yao Y., Wang M., Wang Z., Xue Y., Li B., Ma Y., Shen Y., Wu H. (2024). Recent studies on proteins and polysaccharides-based pH-responsive fluorescent materials. Int. J. Biol. Macromol..

[146] Yadav P., Gupta M., Singh S.P., Parashar P. (2024). Biocompatible Electrospun Hydrogel Fibers for Advanced Wound Healing Therapies. Curr. Pharm. Des..

[147] Gordon A., Li B., Witten J., Nguyen H., Anderson D.G. (2024). Inhalable Dry Powders for Lung mRNA Delivery. Adv. Healthc. Mater..

[148] Naureen F., Shah Y., Rehman M.U., Fazli Nasir F.N., Pirzada A.S., Al-Otaibi J.S., Daglia M., Khan H. (2025). Innovative inhalable dry powder: Nanoparticles loaded with Crizotinib for targeted lung cancer therapy. BMC Cancer.

[149] Rodriguez-Gonzalez R.A., Leung C.Y., Chan B.K., Turner P.E., Weitz J.S. (2020). Quantitative Models of Phage-Antibiotic Combination Therapy. MSystems.

[150] Qureshi K.A., Parvez A., Fahmy N.A., Abdel Hady B.H., Kumar S., Ganguly A., Atiya A., Elhassan G.O., Alfadly S.O., Parkkila S. (2023). Brucellosis: Epidemiology, pathogenesis, diagnosis and treatment-a comprehensive review. Ann. Med..

[151] Shanahan F., Ghosh T.S., O’Toole P.W. (2021). The Healthy Microbiome-What Is the Definition of a Healthy Gut Microbiome?. Gastroenterology.

[152] Cao Z., Sugimura N., Burgermeister E., Ebert M.P., Zuo T., Lan P. (2022). The gut virome: A new microbiome component in health and disease. EBioMedicine.

[153] Ruzzi F., Semprini M.S., Scalambra L., Palladini A., Angelicola S., Cappello C., Pittino O.M., Nanni P., Lollini P.L. (2023). Virus-like Particle (VLP) Vaccines for Cancer Immunotherapy. Int. J. Mol. Sci..

[154] Yue H., Li Y., Yang T., Wang Y., Bao Q., Xu Y., Liu X., Miao Y., Yang M., Mao C. (2025). Filamentous phages as tumour-targeting immunotherapeutic bionanofibres. Nat. Nanotechnol..

[155] Petrov G., Dymova M., Richter V. (2022). Bacteriophage-Mediated Cancer Gene Therapy. Int. J. Mol. Sci..

[156] Wang S.W., Gao C., Zheng Y.M., Yi L., Lu J.C., Huang X.Y., Cai J.B., Zhang P.F., Cui Y.H., Ke A.W. (2022). Current applications and future perspective of CRISPR/Cas9 gene editing in cancer. Mol. Cancer.

[157] Suh G.A., Lodise T.P., Tamma P.D., Knisely J.M., Alexander J., Aslam S., Barton K.D., Bizzell E., Totten K.M.C., Campbell J.L. (2022). Considerations for the Use of Phage Therapy in Clinical Practice. Antimicrob. Agents Chemother..

[158] Dy R.L., Rigano L.A., Fineran P.C. (2018). Phage-based biocontrol strategies and their application in agriculture and aquaculture. Biochem. Soc. Trans..

[159] Miller M., Deiulio A., Holland C., Douthitt C., McMahon J., Wiersma-Koch H., Turechek W.W., D’Elia T. (2020). Complete genome sequence of Xanthomonas phage RiverRider, a novel N4-like bacteriophage that infects the strawberry pathogen Xanthomonas fragariae. Arch. Virol..

[160] Vique G., Mendoza-Barberá E., Ramos-Barbero M.D., Blanco-Picazo P., Sala-Comorera L., Quirós P., Atares S., Salaet I., Muniesa M., Rodríguez-Rubio L. (2025). Efficacy of Erwinia amylovora and Xanthomonas campestris pv campestris phages to control fire blight and black rot in vivo. Microbiol. Spectr..

